# Gene-Length Bias and Compartment Non-Identifiability in Cell-Type Deconvolution of Sheep Skin: A Prospectively Specified and Timestamped Diagnostic Re-Analysis of Wool-Density Transcriptomes in Hetian Sheep

**DOI:** 10.3390/ani16203205

**Published:** 2026-10-12

**Authors:** Mutallip Sattar, Sardar Parhat, Yanping Ma, Gulimire Wufuer, Xiaoxue Zhang, Nana Wei, Jinbo Tian, Rong Gao, Ablat Sulayman, Yuezhen Tian

**Affiliations:** 1 School of Information Management, Xinjiang University of Finance and Economics, Urumqi 830012, China; progfan@aliyun.com (M.S.); sardar312@163.com (S.P.); 2 Key Laboratory of Genetics Breeding and Reproduction of Xinjiang Cashmere and Wool Sheep, National Livestock and Poultry Regional Gene Bank (Xinjiang), Institute of Animal Sciences, Xinjiang Academy of Animal Sciences, Urumqi 830011, China; 17699338428@163.com (Y.M.); gv1mira@163.com (G.W.); zhangxiaoxue0726@163.com (X.Z.); wnn010415@163.com (N.W.); 15299019902@163.com (J.T.); 19155328330@163.com (R.G.); 3 College of Animal Science, Xinjiang Agricultural University, Urumqi 830052, China

**Keywords:** cell-type deconvolution, gene-length bias, identifiability, sheep skin, hair follicle, wool density, pre-registration

## Abstract

Bulk skin transcriptomes cannot distinguish cell-intrinsic gene regulation from shifts in tissue-type composition, and computational deconvolution is widely applied to resolve this limitation. In this study, we performed deconvolution on published Hetian sheep skin RNA-seq data from three high-wool-density and three low-wool-density animals, using the available ovine skin single-cell reference. All hypotheses and analytical workflows were prospectively specified and timestamped prior to estimating cell-type fractions. Our work identifies two major methodological pitfalls for livestock tissue deconvolution. First, gene-length bias distorts deconvolution outputs derived from mixed bulk and 3′ single-cell references. After bias correction, the estimated hair-follicle epithelial fraction changed substantially. This bias cannot be uncovered by standard benchmark workflows, highlighting a hidden failure mode for deconvolution. Second, limited signature separability within the reference prevents reliable discrimination between related epidermal compartments. This non-identifiability strongly disturbs apparent compartment-level effects. When analysed at the granularity the reference reliably supports, we found no compositional difference between high- and low-wool-density groups for the epithelial hypothesis. The dermal hypothesis could not be meaningfully evaluated due to incompatible reference compartment merging requirements. Group-level differential expression remained robust despite this null compositional outcome. We recovered a large set of differentially expressed genes consistent with the original study, including most qPCR-validated candidates. Adjusting for cell-type composition altered part of this gene list, as expected given negligible group-level compositional divergence. Co-expression modules correlated more strongly with inter-individual compositional variation than with wool-density phenotype. This indicates compositional heterogeneity shapes transcriptome co-expression even without measurable mean differences across experimental groups. To avoid misleading biological inference, we propose a set of practical diagnostic checks for deconvolution when developmental or limited single-cell atlases are used for livestock tissues. Collectively, these transcriptomic patterns support cell-state-driven regulation rather than detectable mean shifts in tissue compartment composition.

## Simple Summary

Sheep skin contains many different kinds of cells, and wool grows from structures called hair follicles. When researchers measure gene activity in a whole piece of skin, the measurement mixes together every cell type present, so a difference between two animals can arise either because their cells behave differently or simply because they have different numbers of each cell type. We attempted to separate these two explanations computationally in published data from Hetian sheep with dense and sparse wool. Two things went wrong in instructive ways, and we report both. First, a widely overlooked technical detail—that whole-tissue and single-cell measurements scale differently with the physical length of a gene—distorted our first results severely, misassigning roughly a sixth of the estimated tissue composition and, in the real data, changing the estimate for the main follicle compartment from a few per cent to over four-fifths of the tissue. Second, the only available sheep skin single-cell reference comes from newborn lambs, and in that reference two skin compartments cannot be told apart, so any apparent difference between them is an artefact of the computation rather than a property of the animals. After correcting the first problem and measuring the second, we detected no shift in mean compartment composition between dense-wool and sparse-wool sheep, indicating these transcriptomic patterns are consistent with predominantly cell-state-associated regulation rather than altered cell-type proportions. No animals were used in this study; all data were previously published.

## 1. Introduction

Wool is produced by hair follicles, and the density and arrangement of those follicles set the upper bound on fleece yield in any breed. In sheep, primary follicles form first and give rise to coarser medullated fibres, while secondary follicles form later and produce the finer fibres that dominate commercial wool quality; the ratio between the two is a classical selection target and remains central to contemporary breeding strategy [1,2]. Because follicle number is fixed early in life, understanding the molecular programmes that establish and maintain follicles has direct economic motivation, and transcriptomic profiling of skin has become the standard tool for this purpose [3,4].

A substantial body of literature has investigated sheep skin transcriptomics and fibre-related omics. Bulk RNA sequencing of sheep skin has identified candidate genes for wool fineness. Combined with DNA-methylation data, these analyses have also uncovered candidate loci underlying wool-type variation [5]. Complementary studies have catalogued long non-coding RNAs and circular RNAs involved in follicle growth and cycling [6,7]. Proteomic comparisons across cashmere, guard hair, and wool have further demonstrated distinct structural-protein profiles among fibre types [8]. At the genomic scale, genome resequencing and selective-sweep analyses have mapped genomic regions under selection for wool fineness and broader production-adaptation traits [9,10]. Genome-wide association studies have linked genetic variants to fibre diameter [11], fleece weight, growth, and reproductive performance [12,13]. Single-cell transcriptomics has characterised cellular heterogeneity, resolving epithelial and dermal cell populations during secondary-follicle morphogenesis in fine-wool sheep [14]. Histological work in cashmere goats additionally shows that primary and secondary follicles follow divergent developmental timelines in early post-natal life [15], an important developmental consideration when constructing reference atlases from juvenile animals. Research on keratin-associated protein gene families highlights their large size, tandem organisation, and evolutionary expansion via gene duplication, which creates technical challenges for gene annotation and functional analysis in livestock [16,17,18]. Although these genes are closely associated with fibre structure, their contributions are rarely disentangled from confounding effects originating from variable cellular composition within bulk skin samples.

Two largely separate research domains can be distinguished here: livestock wool-omics focused on fibre phenotypes, and computational methods for transcriptome deconvolution. Bulk skin transcriptome data represent weighted averages across all cell types present in biopsy samples. Observed expression differences between animal groups can arise either from cell-intrinsic gene-expression regulation or from shifts in the proportional representation of constituent cell types. For phenotypes driven by tissue-architecture variation such as follicle density, cell-type compositional change constitutes a biologically relevant hypothesis that is infrequently formally evaluated. Computational deconvolution uses single-cell reference signatures to estimate cell-type fractions from bulk transcriptomes and provides a standard framework to address this issue [19,20,21,22]. Deconvolution performance has been extensively benchmarked for human tissue datasets [23,24].

Nevertheless, applying deconvolution workflows to livestock skin introduces practical challenges that existing human-focused benchmarks only partially cover. Deconvolution accuracy strongly depends on reference quality: unrepresented cell populations can produce non-trivial biases in proportion estimates [25]. Differences in transcript abundance across cell types distort normalisation and mixture decomposition outputs [26], and variable cell size can introduce further systematic errors [27]. Human studies benefit from multiple well-curated reference atlases covering diverse developmental stages and tissues. Equivalent high-quality resources are currently lacking for sheep skin. Consequently, reference suitability cannot be treated as a freely adjustable parameter; its performance must be quantitatively assessed prior to drawing biological conclusions from deconvolution outputs.

The present work builds on published bulk transcriptome and histological data from dense-wool and sparse-wool Hetian sheep reported by Shi et al. [28]. That dataset contains matched histology and bulk transcriptome measurements from identical individuals but does not integrate these two data layers, rendering it suitable for compositional re-analysis. Additional multi-omic investigations of this breed have since been published [29]. We prospectively specified and timestamped two directional hypotheses together with predefined interpretive criteria before performing any cell-proportion estimation. The objectives of this study are as follows: (i) to re-analyse the published bulk transcriptome dataset to test for shifts in follicle-derived cellular compartment proportions between phenotypic groups; (ii) to characterise practical limitations when applying existing deconvolution pipelines to sheep skin bulk data; (iii) to establish diagnostic metrics for evaluating the adequacy of single-cell references for livestock-tissue deconvolution. All results and exploratory observations arising from these analyses are presented in subsequent sections.

In this manuscript, key terms are used with the following working definitions: cell composition/compartment proportion: relative fraction of distinct cell-type compartments within bulk tissue; compositional variation: differences in cell-type fractions between sample groups; cell state: differences in gene-expression profiles within the same cell type; cell-intrinsic regulation: gene-expression changes originating from within a cell rather than shifts in cell-type abundance.

## 2. Materials and Methods

### 2.1. Ethics Statement

This study is a computational re-analysis of previously published, publicly available datasets. No animals were handled, sampled or euthanised by the authors, and no new animal experiments were performed. Ethical approval for the primary data collection was obtained by the original investigators: the skin RNA-seq dataset (NCBI BioProject PRJNA754683) was generated under the approval reported by Shi et al. [28], and the single-cell reference dataset (GEO GSE186204) under the approval reported by its source publication. Accordingly, no additional ethical approval was required for the present study.

### 2.2. Prospectively Specified and Timestamped Analytical Plan

All hypotheses, decision thresholds, and interpretive wording for each potential outcome were documented in a timestamped file prior to deconvolution. The file was hashed (SHA-256 715cd5b6…) and is provided as Supplementary Materials. Two directional hypotheses were defined: hair-follicle epithelial proportion is higher in dense-wool Hetian sheep (HS) than sparse-wool Hetian sheep (HP), and hair-follicle dermal proportion is higher in HS sheep (H2). Three interpretive conditions were predefined: support for the hypothesis, no detectable difference, and opposing trend, each with defined claim strength. This analytical framework avoids post hoc formulation of hypotheses.

### 2.3. Data

Bulk skin RNA-seq comprised six libraries from Hetian sheep, three with dense wool (HS01–03) and three with sparse wool (HP01–03), retrieved from PRJNA754683 and verified by checksum against the ENA file report. The single-cell reference was GSE186204, comprising sheep skin from two animals with contrasting fleece phenotypes. All accessions, checksums, and rationale for excluding other candidate datasets are recorded in the supplementary data inventory.

### 2.4. Quantification

Reads were trimmed with fastp and aligned to the sheep reference assembly ARS-UI_Ramb_v3.0 (GCF_016772045.2) with STAR, and gene-level counts were produced with featureCounts [30]. Strandedness was determined empirically rather than assumed. Annotation assessment for keratin and keratin-associated protein genes was performed during preprocessing; corresponding findings are presented in the Results section.

### 2.5. Reference Panel Construction

Reference cells were assigned to seven compartments—hair-follicle epithelial (HF-epithelial), hair-follicle dermal (HF-dermal), interfollicular epidermis (IFE), fibroblast, endothelial, immune, and a residual muscle/other class—via marker-based scoring of clusters. Cluster assignments were cross-tabulated against published atlas labels to evaluate assignment fidelity. Two reference outputs were generated and carried through all downstream analyses in parallel: one without batch correction and one processed with Harmony correction [31] across the two reference animals. Batch correction of reference datasets has competing rationales, and its influence on deconvolution performance remains incompletely characterised [32,33]. Both variants were retained for all downstream analyses.

### 2.6. Gene-Length Normalisation

Bulk RNA-seq counts scale with transcript length, whereas 3′-end single-cell reference counts approximate one molecule per transcript independent of transcript length. Regressing length-dependent bulk expression against length-insensitive single-cell signatures introduces systematic bias. Bulk counts were converted to TPM-like values using per-gene union-exon lengths derived from the same annotation used for counting, to place bulk and reference inputs onto a length-comparable scale. The magnitude of bias introduced by omitting gene-length correction was quantified within a three-arm controlled experiment, detailed in the Results section. Bias direction depends on the transcript-length distribution of marker genes within each cell compartment.

We further clarify the expected input expression scale for each deconvolution approach used in this work. Some deconvolution algorithms expect raw read counts, while others accept normalised abundance values. Here we adopted a TPM-like transformation as the unified input. This transformation normalises for gene-length differences and sequencing depth simultaneously, making expression values comparable across genes and samples; it provides a consistent input scale that can be reasonably accepted across all deconvolution methods applied in the present study. We use union-exon lengths to avoid isoform ambiguity. Alternative transcript-length definitions yield consistent qualitative deconvolution outcomes with moderate quantitative differences (Supplementary Table S1).

### 2.7. Deconvolution and the Pseudo-Bulk Benchmark

Proportions were estimated using four algorithms with distinct statistical assumptions: MuSiC [20], SCDC [22], BayesPrism [21], and unregularised non-negative least-squares (NNLS). Prior to analysis of experimental data, algorithm performance was benchmarked using 30 pseudo-bulk mixtures generated from held-out reference cells with known ground-truth proportions. Twenty-four mixtures were sampled from a Dirichlet distribution; three matched pairs simulated an approximately 8 percentage-point difference in follicular compartment abundance, matching the effect size implied by source-study histology. Signature and pseudo-bulk mixtures were generated from disjoint sets of reference cells. This benchmark characterised algorithm performance for known effects before application to experimental data.

Pseudo-bulk data inherit transcript-length properties of the reference and cannot capture gene-length-driven bias. A length-perturbed benchmark arm was therefore added for this specific assessment. Three categories of evaluation are distinguished: algorithm benchmarking on pseudo-bulk mixtures, reference identifiability diagnostics, and independent biological validation. No independent biological validation dataset is available in the present study.

To assess sensitivity of deconvolution outputs to gene-length definitions and gene filtering, we performed additional sensitivity analyses. Alternative gene-length definitions including median gene length and transcript-level effective length were tested. We also applied alternative gene-filtering thresholds based on mean expression and detection rate across samples, to examine whether large shifts in estimated follicular epithelial fraction are robust to these parameter choices.

### 2.8. Reference Resolution Diagnostics

Seven annotated compartments do not guarantee seven independently estimable quantities. Compartment separability was evaluated with three complementary diagnostics. First, pairwise signature profile correlations were calculated for all compartment pairs, and the count of genes highly and uniquely expressed within each compartment was recorded. Second, identifiability failure was quantified by iteratively shifting modelled abundance between compartment pairs and recording corresponding increases in residual fitting error. A threshold of correlation > 0.85 and a residual error increase < 5% under 20% mass transfer defines high collinearity with negligible fitting cost, indicating non-identifiable compartment pairs. Third, deconvolution was repeated across 50 random 50% subsamples of reference cells to detect correlated or mirror-image behaviour between compartment pairs.

Pairs failing these diagnostics were merged following predefined criteria: high signature collinearity together with absence of exclusive marker genes. Results are reported both for the original seven-compartment and merged granularity to illustrate merging effects. Because merging alters biological interpretation of compartments, differences derived from merged outputs were decomposed back into original compartments. Hypothesis testing was always performed against compartments defined in the prospectively specified analytical plan.

### 2.9. Differential Expression and Composition Adjustment

Differential expression was computed with DESeq2 [34], applying the threshold from the original source study and an additional stricter threshold. Composition-aware analysis included inferred abundance of the dominant compartment as a model covariate. Sequencing QC revealed insert-size differences between experimental groups. Because covariates correlated with group assignment can alter differential-expression results regardless of biological origin, null covariates with matching collinearity were constructed. Observed effects from real and null covariates were compared, rather than directly interpreting covariate-driven fold-change attenuation. Observations related to insert-size bias are presented within Results.

### 2.10. Co-Expression Network Analysis

Weighted gene co-expression network analysis (WGCNA) was performed on the bulk-expression matrix. Module association was tested against phenotype label and inferred compartment proportions, which were treated as continuous traits. Counts were transformed following published recommendations for RNA-seq-derived co-expression networks [35]. The multi-trait analytical design follows multiWGCNA implementation [36]. Soft-threshold selection outcomes and related decision-making are reported in Results. With six samples, module-trait correlations are treated as hypothesis-generating outputs and are not interpreted as predictive evidence. Consistency between outputs from the two reference-processing variants represents sensitivity-analysis agreement rather than independent biological replication.

### 2.11. Reproducibility

All analytical stages logged outputs, intermediate files and random seeds within a versioned directory. Each workflow stage included predefined pass-fail acceptance criteria. The global random seed for all stochastic computations was set to 20260817.

The differential-expression model formula was ~ group (HS versus HP), fitted in DESeq2; the Benjamini–Hochberg procedure was used for multiple-testing adjustment. Prospectively specified and timestamped hypotheses H1 and H2 were evaluated with two-sided Wilcoxon rank-sum tests supplemented by Cliff’s δ effect-size estimation; one-sided testing was predefined in the analytical plan due to directional predictions for compartment proportions in dense-wool animals. Reference signatures were built from log-normalised single-cell profiles, with two parallel processing variants (uncorrected and Harmony batch-corrected).

Union-exon gene lengths derived from genome annotation were used for gene-length normalisation. Exact software and package versions are documented in the dedicated reproducibility supplementary document.

For WGCNA co-expression analysis, outputs are explicitly exploratory descriptive results. Consistency observed across the two reference-processing variants is interpreted as a sensitivity-analysis outcome, not independent biological replication.

Intermediate outputs including source values for each figure panel, a computational environment snapshot, and a file-hash manifest are provided as Supplementary Materials, enabling full reproduction of the reported quantities without re-running read alignment.

## 3. Results

### 3.1. Algorithmic Validation and a Normalisation Error That Validation Missed

**Figure 1:**
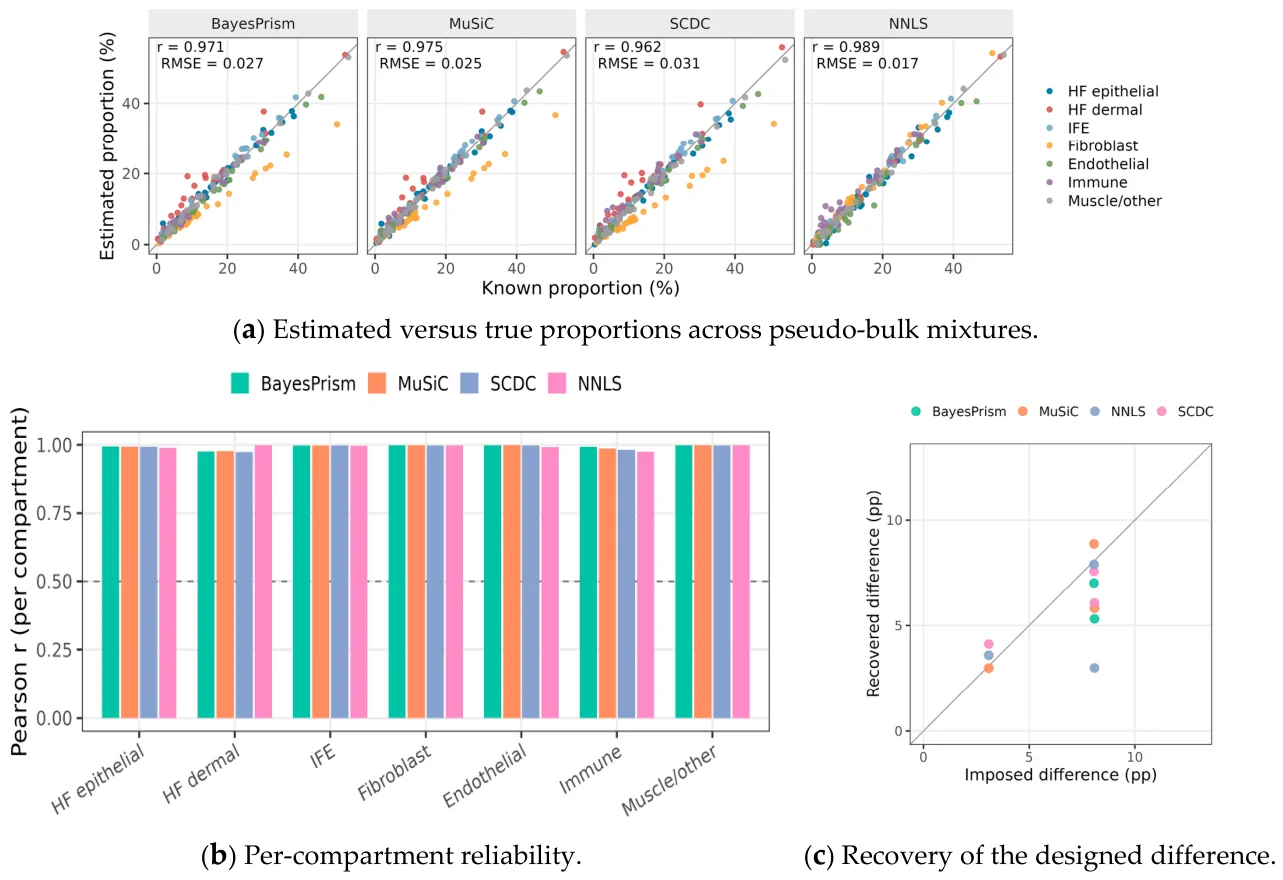
Algorithmic validation on pseudo-bulk mixtures with known composition. (**a**) Estimated versus true compartment proportions across all pseudo-bulk mixtures; the diagonal marks perfect re-covery. (**b**) Per-compartment reliability, given as the correlation between estimated and true proportion for each cell type separately. (**c**) Recovery of the designed group difference, comparing the effect size built into each mixture with the effect size recovered by each algorithm. All algo-rithms recover known proportions accurately, yet this benchmark cannot detect the gene-length bias quantified in Table 1, because pseudo-bulk inherits the reference’s own 3’ bias so that the error cancels on both sides of the regression. Points represent individual pseudo-bulk mixtures; lines denote algorithms.

Across 30 pseudo-bulk mixtures with known composition, all four algorithms recovered proportions accurately, with overall correlations between estimated and true values of 0.962–0.989 without batch correction and 0.938–0.962 with Harmony correction, and with the sign of the designed compartment difference recovered in all twelve algorithm–pair combinations (Figure 1). By the usual standard, this would licence proceeding to the real data. It did not, and the reason is instructive.

Bulk counts scale with transcript length; the 3’-end single-cell reference does not. The genes distinguishing the compartments of interest differ systematically in length—collagens such as COL1A1 (5912 bp) and DCN (4858 bp) are long, whereas hair keratins such as KRT25 (1656 bp) and KRT27 (1601 bp) are short—so the resulting bias is not merely large but directional along precisely the axis this study concerns. A three-arm controlled experiment quantified it (Table 1): unbiased pseudo-bulk gave r = 0.990 with hair-follicle epithelial bias of −1.58 percentage points (pp); multiplying counts by transcript length collapsed recovery to r = 0.399 and inflated the bias to −17.32 pp; dividing the same data back by length restored the original values exactly. On the real data, correcting this changed the estimated hair-follicle epithelial fraction from 3.3% to 81.4% (MuSiC algorithm, no-Harmony reference variant, group mean across six study samples, unmerged hair-follicle-epithelium compartment only), the latter being the value consistent with follicle-dense skin. These values are model-derived deconvolution estimates and not direct histological measurements.

The reason the benchmark passed while the analysis was invalid is general and worth stating plainly: pseudo-bulk is summed from the reference’s own cells and therefore carries the identical 3’ bias, which cancels on both sides of the regression. Only real bulk data can expose the error. Pseudo-bulk validation, on its own, cannot establish that a deconvolution pipeline is correctly normalised.

We carried out sensitivity analyses using alternative gene-length definitions and alternative gene-filtering strategies. The large magnitude change in estimated follicular epithelial cell fraction after gene-length correction was partially preserved across several alternative settings, yet we also observed quantitative deviations under some filtering and length-definition choices. These sensitivity outcomes are provided in Supplementary Materials.

### 3.2. Two Compartments in the Reference Are Not Separately Identifiable

The seven annotated compartments do not correspond to seven measurable quantities. Hair-follicle epithelium and interfollicular epidermis have signature profiles correlating at r = 0.964, and interfollicular epidermis possesses no genes expressed highly and exclusively relative to follicular epithelium; only two of twenty-one compartment pairs were separable by our criterion at all. Three independent diagnostics then established that the failure is one of identifiability rather than precision.

**Figure 2:**
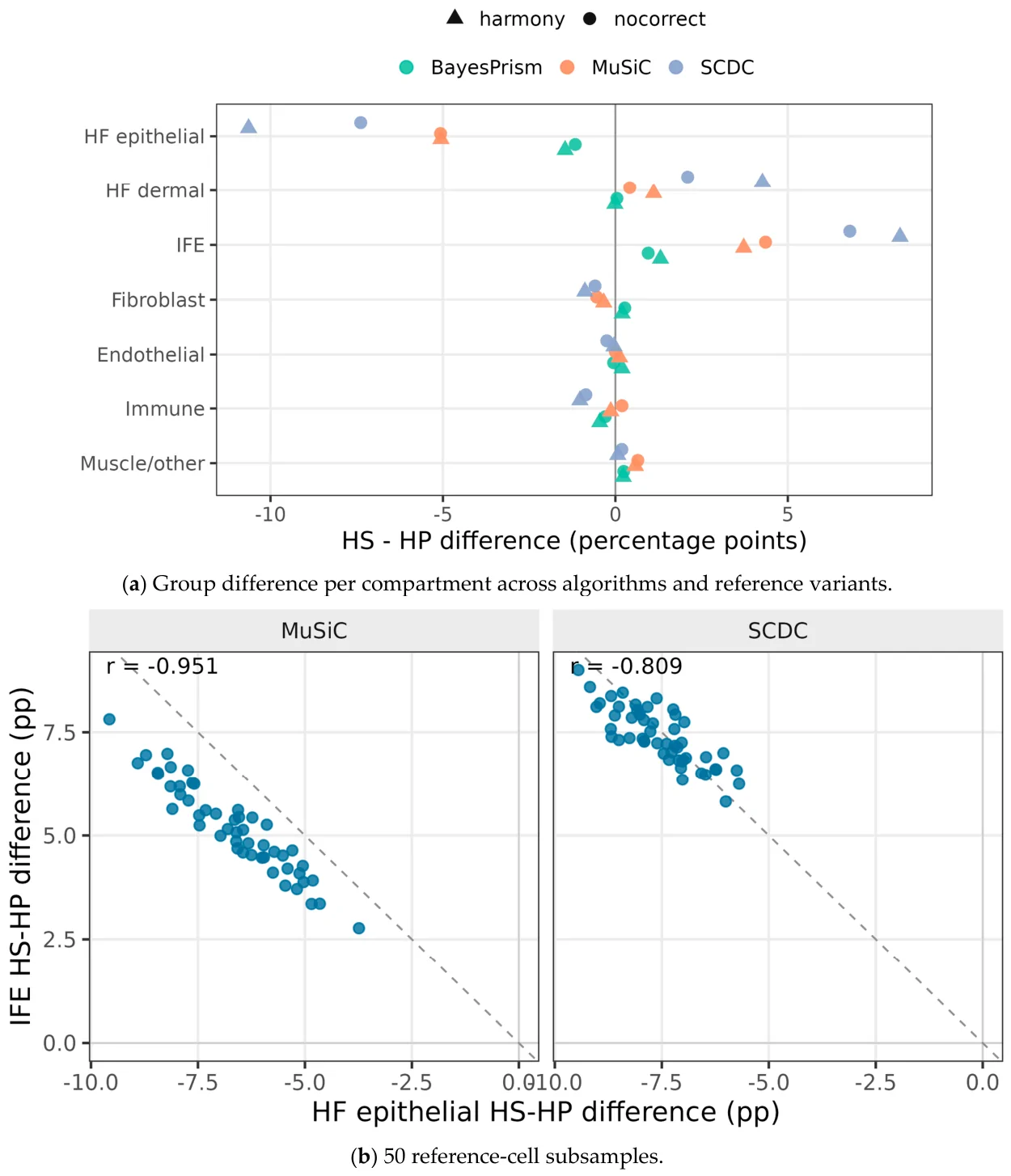
Robustness of the compositional estimates and the identifiability limit. (**a**) Group difference per compartment across all algorithm and reference-variant combinations, showing the sign and magnitude of every estimate obtained under the registered analysis branches. (**b**) Mirror behaviour of hair-follicle epithelium and interfollicular epidermis across 50 reference-cell subsamples; each point is one subsample, and a slope approaching −1 indicates that the model is redistributing a single epithelial pool between two labels rather than measuring two independent quantities. The mirror-image variation between follicular epithelium and interfollicular epidermis reflects numerical redistribution of non-identifiable compartments and does not represent a true biological inverse relationship.

First, moving mass from hair-follicle epithelium to interfollicular epidermis is essentially free: relative to the unshifted fit, a 2 pp shift changes the residual error by −0.003%, and even a 20 pp shift raises it by only 0.23% (Table 2). The penalty is not merely small but almost flat in the size of the shift, which is the signature of a direction in parameter space that the data do not constrain. Second, across 50 random subsamples of reference cells, the estimated group differences for the two compartments moved as mirror images, correlating at r = −0.95 (MuSiC) and r = −0.81 (SCDC) without batch correction, and r = −0.95 and r = −0.72 with it (Table 3, Figure 2). Individually, each compartment swung by 4.6 to 10.9 pp; their sum was stable. Third, in the leave-one-compartment-out jackknife, removing interfollicular epidermis alone eliminated 76.7–95.2% of the apparent follicular effect, whereas removing any other compartment left it largely intact.

### 3.3. Neither Registered Hypothesis Is Supported

**Figure 3:**
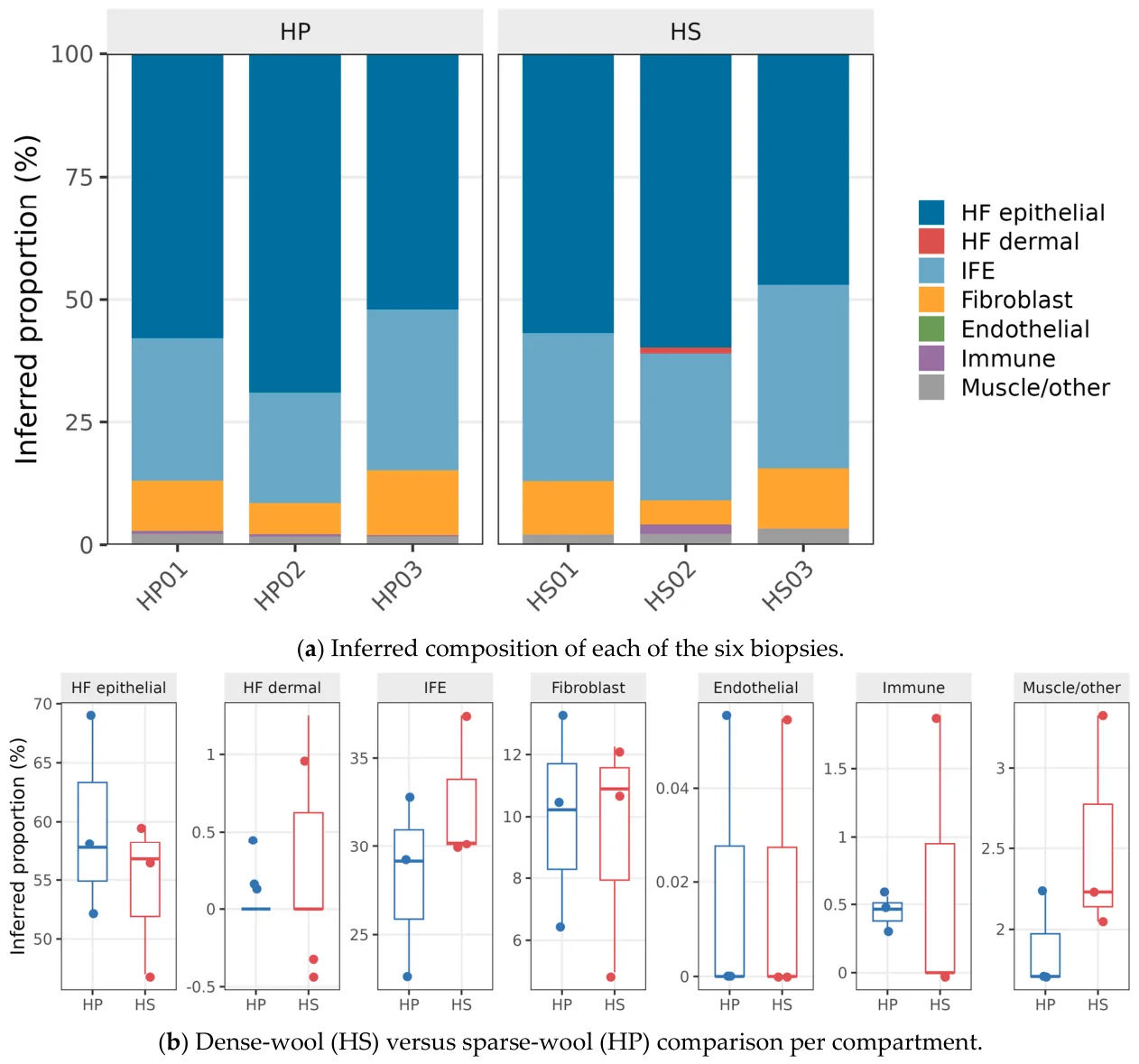
Inferred cell-type composition and the group comparison. (**a**) Inferred composition of each of the six biopsies, with each bar giving the full compartment breakdown for one animal. (**b**) Dense-wool (HS, dense-wool Hetian sheep) versus sparse-wool (HP, sparse-wool Hetian sheep) comparison per compartment, showing group means with the spread of individual estimates. Follicular epithelium dominates every biopsy, and the between-group differences are small relative to the variation between individuals within each group.

Re-tested at the granularity the reference supports (Figure 3), the three model-based algorithms agree almost exactly (pairwise r > 0.998), confirming that their earlier disagreement was produced by the unidentifiable split rather than by the algorithms themselves. Non-negative least squares remains an outlier, forcing the dermal compartment to 0%, which is the expected behaviour of an unregularised fit on collinear signatures and is reported as a floor rather than as a co-equal estimate.

Against this background, H1 is not supported (Table 4). Epithelial proportion differs by −0.21 to −0.72 pp without batch correction and −0.15 to −2.38 pp with it in both cases in the direction opposite to the registered prediction. No test approaches significance; with three animals per group, Cliff’s δ cannot exceed modest values, and the smallest one-sided *p*-value for the epithelial comparison is 0.65.

H2 requires a more careful statement, because the merge that makes the epithelial test valid does not preserve the quantity H2 was written about. The registered H2 concerns follicle-inductive mesenchyme, that is HF_dermal (dermal papilla and dermal sheath); the merged Dermal compartment additionally contains dermal fibroblasts, for which the pre-registration explicitly declined to predict a direction. Decomposing the merged difference shows that these two constituents do not behave as one quantity (Table 5). In five of six variant/algorithm combinations, they carry opposite signs, so the merged value is a partial cancellation rather than an aggregation, and its magnitude is not attributable to the registered compartment. HF_dermal itself is estimated at 0.006–4.26% depending on algorithm, with three of six estimates placing at least one group at exactly zero—the behaviour expected of a compartment that the reference cannot support rather than of a measured quantity. We therefore report H2 as not evaluable with this reference: the merged test that is statistically valid does not isolate the registered compartment, and the unmerged compartment that matches the registered definition is not identifiable. The trend previously noted in the merged value—positive in 94–100% of subsampling repeats but with its sign broken by the Harmony jackknife (range −0.58 to +5.48 pp)—is reported for completeness and is driven predominantly by fibroblasts, which were not part of any registered prediction.

The interpretation is that the bulk transcriptomic difference between dense- and sparse-wool animals is not explained by a shift in mean cell-type composition at any granularity this reference can resolve. This does not contradict the histological difference reported by Shi et al. [28]; follicle counts and biopsy composition are distinct quantities, and a real difference in the former need not produce a detectable difference in the latter within a fixed-area sample. It does, however, redirect attention from cell number to cell state.

### 3.4. Differential Expression Is Unaffected and Independently Corroborated

Differential expression does not depend on the compositional result and stands on its own. Of 17,064 tested genes, 1811 were differentially expressed at the source study’s threshold (926 up, 885 down) and 785 at the stricter threshold, against 1452 reported by the source study—a ratio of 1.25, indicating concordant scale rather than a discrepancy requiring explanation. Seven of the eight genes that the source study validated by qPCR are quantifiable in our data, and six of those seven reach significance (INHBA does not, adjusted *p* = 0.41), with effects ranging from modest (MAP2K2, log_2_FC = 0.455) to large (KIT, log_2_FC = −3.365, adjusted *p* = 1.2e − 29). Recovering the same genes as differentially expressed in an independent reprocessing of the raw reads is a consistency check on our pipeline; we deliberately do not claim directional concordance with the published qPCR, because the source study’s per-gene fold-change directions were not extracted into our archived tables and we decline to assert a comparison we cannot document.

**Figure 4:**
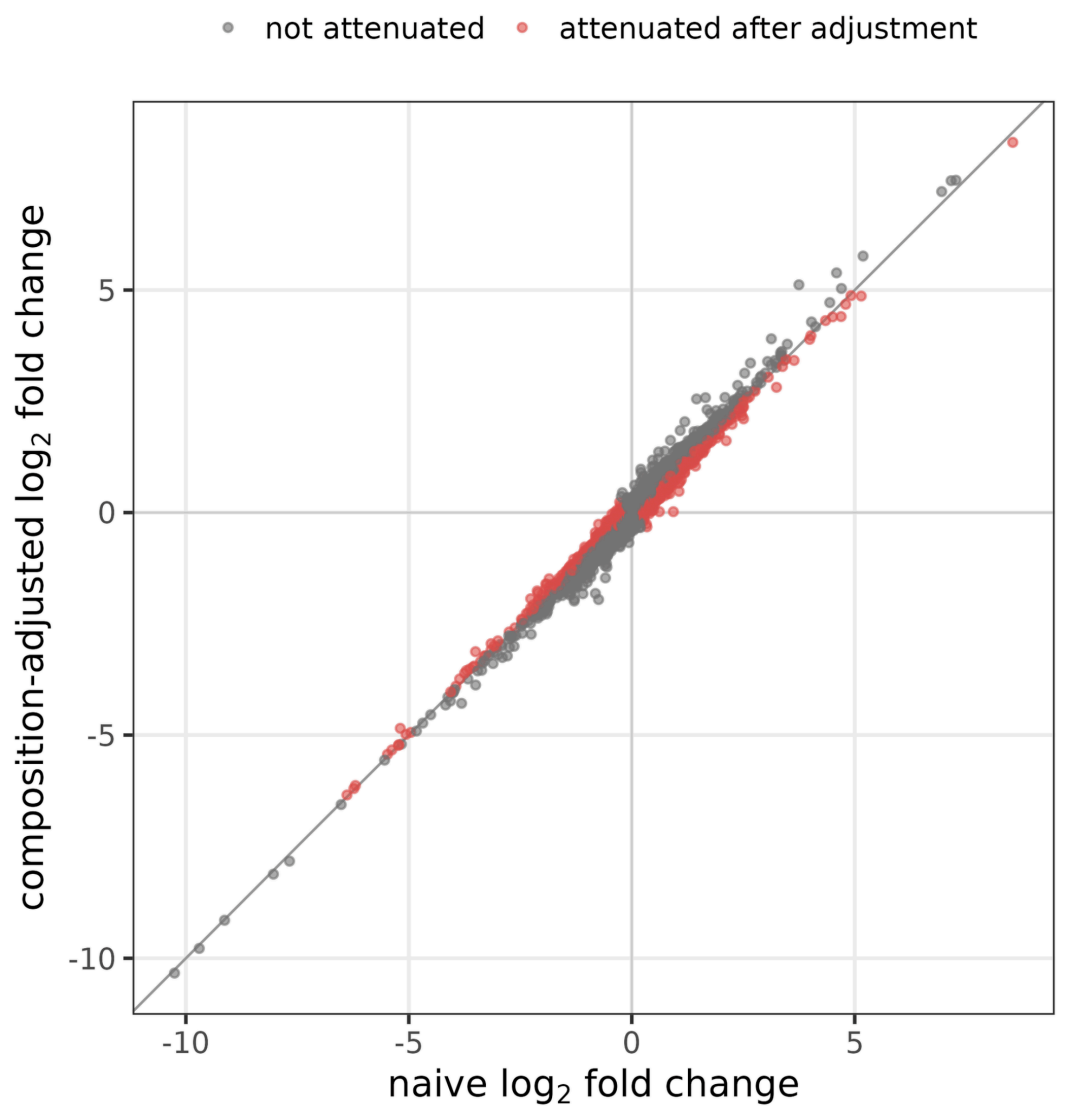
Log2 fold changes before and after adjustment for inferred composition. The near-identity relationship (r = 0.982) is the expected consequence of the compositional null: if mean composition barely differs between groups, adjusting for it cannot substantially alter the differential expression result. Each point represents one gene.

Adding inferred composition as a covariate retained 87.6% of differentially expressed genes (88.9% under Harmony) with log_2_ fold changes correlating at 0.982 before and after adjustment (Figure 4), although 35.7% of all genes were attenuated to some degree. Adjustment is not purely subtractive: of the 1811 unadjusted genes, 1610 were retained and 201 lost, while 433 genes became newly significant, for an adjusted total of 2043. We report the gained set alongside the retained fraction because quoting retention alone would understate how much the covariate reorganises the result. These observations are mutually reinforcing rather than redundant: if mean composition barely differs between groups, adjusting for it should barely change the differential expression result, and it does not.

A quality-control flag required a controlled test rather than direct interpretation. Dense-wool libraries had shorter inserts than sparse-wool libraries, and adjusting for insert size reduced the gene count from 1811 to 334 (18.4%), which appears alarming. However, random covariates constructed to have the same collinearity with the group label (|r| ≈ 0.77) produced a median of 383 genes (21.1%), placing the real covariate squarely inside the null distribution, while covariates with lower and higher collinearity produced 1443 and 91 genes, respectively. The reduction therefore reflects collinearity with group, and we cannot fully disentangle potential independent technical effects of insert size given our small sample size; we report this as an important limitation. Without this control, the same observation would have supported a false alarm.

The annotation audit yielded a quantitative side-result. Of 148 keratin-family genes in the assembly, 95 carry only LOC identifiers; among 42 differentially expressed keratin-family genes, 35 are LOC-only. A symbol-based analysis would therefore miss 83.3% of the keratin-family signal, which is consistent with the known annotation difficulties of these tandemly arrayed families [17,18] and explains how a caprine-labelled KRTAP12-1 entry could propagate into a sheep gene table.

### 3.5. Composition Organises Co-Expression More Strongly than the Phenotype Label

**Figure 5:**
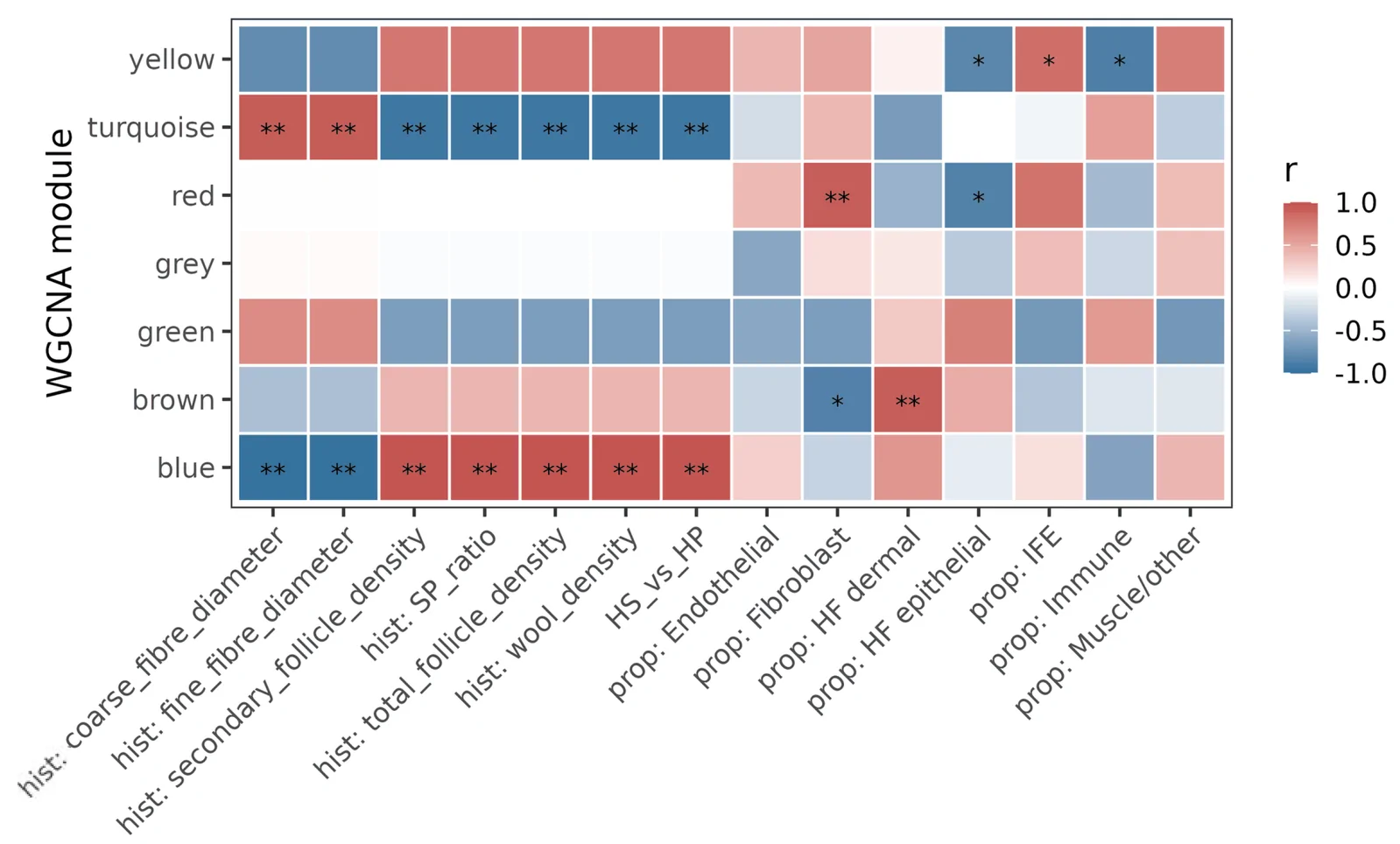
Module–trait correlation structure. Columns are the inferred composition traits together with the dense/sparse phenotype label; in five of the seven modules, a composition trait outranks the label.

Network analysis produced seven modules with 0.3% of genes unassigned. In five of the seven, an inferred composition trait correlated more strongly with the module eigengene than the dense/sparse label did, and this held in both reference variants (Figure 5, Table 6). This is coherent with the null above rather than contradictory: mean composition does not differ between groups, yet composition varies between individuals, and that variation structures co-expression. Co-expression analysis of sheep skin has previously been used to nominate follicle-development candidate genes from the phenotype contrast alone [37]; our result indicates that in these data the compositional axis is the stronger organising variable. With six samples, these correlations are hypothesis-generating, and we present them as such. These numerical r and *p* values are descriptive only and do not prove stable biological associations.

## 4. Discussion

The result we set out to obtain did not materialise, and the reasons it did not are more transferable than the result would have been. Three findings deserve emphasis.

The gene-length bias is the most immediately actionable. It is not a subtle statistical concern but a first-order error: uncorrected, it displaced 17.3 pp of the compositional estimate and reduced the recovery correlation from 0.990 to 0.399, and it did so directionally along the follicular–dermal axis because the marker genes of those compartments differ systematically in transcript length. The mechanism is elementary—bulk counts scale with length, 3’-end single-cell counts do not—and precisely because it is elementary, it is easy to omit when a pipeline is assembled from tools that each behave correctly in isolation. Our third experimental arm, which recovers the original values exactly upon dividing the perturbation back out, establishes transcript length as the cause rather than a correlate. The related observation that transcriptome size varies across cell types and distorts both normalisation and deconvolution [26] concerns a different quantity but points the same way: the assumption that bulk and single-cell measurements are on comparable scales requires verification, not faith.

The secondary lesson concerns validation practice. Our pseudo-bulk benchmark passed at r > 0.96 on 30 mixtures while the pipeline applied to real data was invalid because pseudo-bulk constructed from reference cells inherits the reference’s length characteristics and the bias cancels on both sides of the regression. This is a structural blind spot, not bad luck, and it applies to any benchmark built this way. Benchmarking studies have examined the influence of transformation, normalisation, and marker selection on deconvolution accuracy [23,24], and have shown that measured accuracy depends strongly on the testing environment in which it is assessed [38], but a benchmark whose synthetic mixtures share the reference’s technical idiosyncrasies cannot detect mismatches between the reference and the real target. The remedy we adopted—deliberately perturbing the property in question and confirming that the benchmark responds—is cheap and should be routine.

The identifiability finding is the one most likely to affect other livestock studies. Annotating seven compartments does not create seven measurable quantities, and the gap between annotation and estimability was large here: signature collinearity of 0.964, no exclusive markers for interfollicular epidermis, a residual-fit penalty of only 0.23% for relocating 20 pp of mass, mirror-image behaviour at r = −0.72 to −0.95 across subsamples, and loss of 76.7–95.2% of the apparent effect when interfollicular epidermis alone is dropped. Any one of these could be dismissed; together they establish that the seven-class split was manufacturing an effect by arbitrarily partitioning a single pool. Had we reported the seven-class result without these checks, we would have published an apparent 7 pp shift in follicular epithelium that dissolves on merging. We prioritised merging follicular epithelium and interfollicular epidermis because this pair drove the spurious apparent treatment-associated compositional shift, despite poor separability across most other compartment pairs. Developmental stage mismatch between the lamb-skin reference and adult-skin bulk samples is a plausible contributing factor to this limited resolvability, alongside other constraints including limited donor number, cell-state heterogeneity, sequencing chemistry, cell capture efficiency, and marker selection; our analysis cannot disentangle their individual contributions. Livestock species typically have one atlas per tissue, generated at whatever developmental stage the original study required, and reusing it for a different stage silently changes what the compartment labels mean. Concerns about missing reference populations [25] and about cell-size heterogeneity [27] belong to the same family of problems: the reference is not a neutral instrument.

Our null on composition should be read narrowly and precisely. We do not contest the histological difference reported by Shi et al. [28]; their follicle-density measurements stand, and our data cannot address them. What we can say is that within these six biopsies, the bulk expression difference is not accompanied by a detectable difference in mean compartment proportions at any granularity the available reference supports. Follicle density per unit skin area and cell-type proportion within a biopsy are related but distinct quantities, and the former can differ while the latter does not, particularly when follicular epithelium already constitutes the great majority of the tissue—a ceiling effect that limits how much a further increase in follicle number can alter proportions. Given the limited statistical power at n = 3 per group, the present data are compatible with cell-state-associated regulation, but they cannot confirm this mechanism, identify which cell types are affected, or exclude unmeasured compositional shifts (r = 0.982 between adjusted and unadjusted fold changes, 87.6% of genes retained). Direct confirmation would require adult single-cell or spatial profiling of the same biopsies. This shifts the mechanistic question from how many follicular cells are present to what those cells are doing, a question that spatial or single-cell profiling of adult skin from the same animals could address directly and bulk profiling cannot.

As a purely exploratory observation, the co-expression result provides non-validating descriptive insight for our deconvolution outputs. Importantly, since module detection and compositional estimates use the same bulk matrix, this association cannot constitute independent supporting evidence. With only six samples, module structures are inherently unstable; we therefore treat these correlations as descriptive patterns without strong inferential conclusions. It nonetheless suggests that treating inferred composition as a continuous covariate may be informative for livestock skin study design.

Two further methodological points bear on how such studies should be reported. First, the insert-size episode illustrates that with n = 6 almost any covariate correlated with the group label will substantially reduce detected differential expression, so the reduction itself is uninformative; only comparison against matched-collinearity null covariates distinguishes a real confounder from an artefact of collinearity, and in our case it was the latter. Second, the annotation audit showing that 83.3% of differentially expressed keratin-family genes carry only LOC identifiers is a reminder that in livestock genomes the analysis is bounded by annotation quality. Studies restricted to approved symbols are not conservative; they are systematically blind to the gene families most relevant to fibre traits [16,17,18].

Taken together, these results argue for a specific ordering of work in deconvolution studies of livestock tissue: verify that bulk and reference measurements are on a common scale by perturbation rather than assumption; measure the granularity at which the reference can support claims before formulating claims at that granularity; and report the resulting resolution limit as a finding rather than concealing it through a granularity choice made after seeing the results. Pre-registration made this ordering enforceable in our case, since the hypotheses and permitted wording were fixed before any estimate existed.

### Limitations

The constraints on this study are substantial, and we state them without mitigation.

The sample size is six animals, three per group. This is the size of the published dataset and cannot be increased retrospectively. It has three consequences that recur throughout our results: non-parametric tests on three versus three cannot produce small *p*-values regardless of effect size, so our null on composition is a statement about undetectability at this sample size rather than a demonstration of equivalence. Conversely, a biologically meaningful compositional change could remain statistically undetectable, and readers should not interpret the null as evidence of absence. For 3-versus-3 comparisons, Cliff’s δ can mathematically reach ±1 but falls on a coarse discrete grid with high estimation uncertainty; our observed effect sizes remained modest, statistical resolution and precision are severely limited by the small sample size, and any covariate correlated with the group label sharply reduces detected differential expression, as our matched-collinearity control confirmed. Readers should treat the compositional null as “no detectable difference” and not as evidence of no difference.

The reference atlas is the binding constraint on resolution. It derives from three-day-old lamb skin in anagen, while the bulk data come from adult animals, and no alternative ovine skin atlas exists. This developmental mismatch is a plausible contributing factor to the identifiability failure we document, of the implausibly small fibroblast fraction, and of the absence of a distinguishable interfollicular population. Our merged-granularity results are valid within this limit but cannot recover information the reference does not contain. Moreover, the developmental mismatch may systematically assign adult cells to imperfectly matching compartments; merging collinear labels improves identifiability but does not guarantee that the proportions within merged compartments are unbiased. Should an adult sheep skin atlas become available, the seven-compartment question we were forced to abandon becomes answerable, and our diagnostics provide the test for whether it has become so.

One registered hypothesis could not be tested as written. H2 named the follicle-inductive mesenchyme, but that compartment is not identifiable in this reference, and the merge required to obtain a valid test folds it together with dermal fibroblasts, which the pre-registration excluded from directional prediction. We report H2 as not evaluable rather than substituting the merged quantity for the registered one; a reader interested in the merged trend will find it in Table 5, decomposed so that its origin is explicit.

Our composition estimates are inferred, not measured. We have no matched histology or flow cytometry for these six animals, so the proportions cannot be validated against ground truth in the samples where they matter; the pseudo-bulk benchmark validates the algorithms under controlled conditions, not the accuracy of adult-skin estimates. Direct validation would require matched histology or independent single-cell data from the same adult biopsies. Relatedly, the histological follicle counts of the source study were obtained from the same animals but are not paired with our compartment estimates in any way that would licence a direct comparison, and we have deliberately avoided presenting such a comparison.

Several technical approximations remain. The reference signature is constructed from log-normalised expression, which is standard practice but not scale-exact for a linear mixture model. Our gene-length correction uses union-exon lengths rather than expression-weighted effective transcript lengths, which is the right correction at the gene level but ignores isoform usage differences between compartments. Batch correction of the reference is defensible in either direction, and we report both variants throughout rather than resolving the question; where they disagree, as in the jackknife stability of the dermal trend, we report the disagreement.

Our sensitivity analyses indicate that the strong shift in estimated follicular-epithelial proportion induced by gene-length correction is not fully invariant to gene-length definitions and gene-filtering criteria. While the qualitative influence of gene-length bias persists across many reasonable pre-processing configurations, quantitative fraction estimates show visible variation under alternative parameter choices. This further underscores that absolute cell-type fractions derived from deconvolution should be interpreted with caution.

Finally, co-expression modules and their compositional associations are exploratory. With n = 6, WGCNA yields unstable module structures. Moreover, both module and deconvolution outputs originate from the same bulk transcriptome matrix; hence their association cannot be interpreted as independent validation. We report this consistent, interpretable pattern without claiming cross-cohort replicability. Correlations derived from six-sample data are sensitive to individual animals.

Another important limitation originates from our single-cell reference dataset. There exists a developmental mismatch between the reference cells and bulk RNA-seq samples used in the present study, together with limited cell-type resolution within the reference. Even after merging non-identifiable epithelial compartments, absolute cell-type proportions estimated from real bulk data should be interpreted with caution. Such reference-related constraints cannot be fully eliminated solely by compartment merging and may still bias quantitative estimates of skin deconvolution. We note that this limited resolution may arise from multiple interrelated reference properties (developmental stage, donor number, cell-state distribution, sequencing chemistry, capture method, and marker selection), and our data cannot isolate the contribution of any single factor.

## 5. Conclusions

Deconvolution of bulk livestock skin transcriptomes against a single-cell reference requires two verifications that are easy to omit and consequential when omitted: that both measurements are on a length-comparable scale and that the annotated compartments are separately identifiable given the reference actually available. In Hetian sheep skin, failing the first displaced up to 17.3 pp of the compositional estimate and reduced recovery of known proportions, while failing the second manufactured an apparent 6–11 pp shift in follicular epithelium that dissolves once two collinear compartments are merged. After both corrections, and with hypotheses registered in advance, the dense- versus sparse-wool expression difference is not accompanied by a detectable shift in mean cell-type composition, while differential expression remains robust and recovers the genes the source study validated by qPCR. Exploratory co-expression analysis reveals a descriptive pattern: inter-individual compositional variation organises transcriptome structure more strongly than the wool-density phenotype label. Within this dataset, this observed pattern renders cell-state-associated effects a plausible interpretation; nevertheless, an absence of detectable mean-composition differences cannot rule out subtle shifts, changes in rare cell populations, or variation within merged compartments, and definitive biological interpretation is constrained by sample size and reference limitations. We offer the length-bias control, the separability diagnostic, and the subsampling mirror test as prerequisites for compositional inference in tissues where only a single, developmentally mismatched reference atlas exists. We further caution that absolute cell-type proportion estimates remain subject to reference-derived limitations even after compartment merging and are also sensitive to choices of gene-length definitions and gene-filtering thresholds.

## Figures and Tables

**Table 1:** Three-arm controlled test of gene-length bias. The third arm confirms that the corruption is exactly invertible, establishing transcript length as its cause rather than a confound.

Arm	*r*	RMSE	HF Epithelial Bias (pp)
Unbiased pseudo-bulk	0.990	0.017	−1.58
Counts × transcript length	0.399	0.235	−17.32
Same, divided back by length	0.990	0.017	−1.58

**Table 2:** Cost of relocating mass between the two epithelial compartments, relative to the unshifted fit. A nearly flat penalty curve indicates that the split is not constrained by the data.

Shift (pp)	RMSE	Change (%)
0	434.473	0.000
2	434.459	−0.003
5	434.486	0.003
10	434.655	0.042
20	435.462	0.228

**Table 3:** Mirror behaviour of the two epithelial compartments across 50 reference-cell subsamples. Taken alone, each compartment shows a large group difference; merged, the effect nearly vanishes. The final column shows the one weak signal that survives merging.

Variant	Algorithm	r (HF Epi, IFE)	HF Epi Alone (pp)	Merged Epi. (pp)	Merged Dermal (pp)	Dermal % > 0
No correction	MuSiC	−0.951	−6.66	−1.48	0.51	94
No correction	SCDC	−0.809	−7.55	−0.15	1.13	100
Harmony	MuSiC	−0.948	−6.19	−1.63	0.91	100
Harmony	SCDC	−0.717	−10.90	−2.56	3.87	100

**Table 4:** Hypothesis tests at merged granularity. H1 predicted a positive epithelial difference. Values are percentage-point differences (HS, dense-wool Hetian sheep minus HP, sparse-wool Hetian sheep); no test reaches significance. The merged Dermal column is shown for completeness but does not isolate the compartment named by H2, which is decomposed in Table 5. NNLS (non-negative least-squares) is shown as an unregularised floor; excluding it, the epithelial range is −0.15 to −2.38 pp (percentage points).

Algorithm	No Correction		Harmony	
	Epithelial	Dermal	Epithelial	Dermal
BayesPrism	−0.21	0.32	−0.15	0.18
MuSiC	−0.72	−0.13	−1.34	0.77
SCDC	−0.59	1.51	−2.38	3.38
NNLS	7.63	0.00	7.64	−0.67
Minimum one-sided p	0.65	0.35	0.65	0.20

**Table 5:** Decomposition of the merged dermal difference into its two constituents. HF_dermal is the compartment named by the registered H2; fibroblast was explicitly excluded from directional prediction. Opposite signs within a row mean the merged value is a cancellation, not an aggregate. Values are percentage-point differences (HS, dense-wool Hetian sheep minus HP, sparse-wool Hetian sheep).

Variant	Algorithm	HF_dermal	Fibroblast	Merged Dermal
No correction	BayesPrism	0.044	0.273	0.317
No correction	MuSiC	0.417	−0.544	−0.127
No correction	SCDC	2.096	−0.591	1.506
Harmony	BayesPrism	−0.016	0.197	0.181
Harmony	MuSiC	1.110	−0.339	0.770
Harmony	SCDC	4.264	−0.884	3.380

**Table 6:** Modules whose eigengene correlates more strongly with an inferred composition trait than with the phenotype label. Reproduced in both reference variants. Note: *p*-values are descriptive-exploratory and do not represent formal inferential statistics due to small sample size (n = 6).

Module	Best Composition Trait	r	*p*
MEred	Fibroblast	0.94	0.006
MEbrown	HF dermal	0.92	0.009
MEgrey	Endothelial	−0.92	0.010
MEyellow	IFE	0.86	0.029
MEgreen	HF epithelial	0.75	0.085

## Data Availability

All primary data analysed in this study are third-party and publicly available. Bulk skin RNA-seq data are available from the NCBI Sequence Read Archive under BioProject accession PRJNA754683, comprising six libraries from Hetian sheep (three dense-wool, three sparse-wool); these data were generated by Shi et al. [28]. The single-cell RNA-seq reference used to build the deconvolution signature is available from the NCBI Gene Expression Omnibus under accession GSE186204. The reference genome assembly and annotation are available from NCBI under accession GCF_016772045.2 (ARS-UI_Ramb_v3.0). Two candidate datasets were assessed and excluded, and we record the reasons so that the selection is auditable. An additional ovine skin single-cell dataset was excluded because its metadata did not permit unambiguous assignment of cells to follicular versus interfollicular compartments, which is the distinction on which this study turns. A caprine skin single-cell dataset was excluded because cross-species reference transfer would have introduced an orthology-mapping error term that cannot be separated from the compositional effect being estimated with six bulk samples; this exclusion is a deliberate methodological restraint rather than a data-availability limitation. No other skin single-cell dataset for Ovis aries was identified at the time of analysis, which is the reason the resolution limit reported here could not be circumvented by substituting a better-matched reference. The analysis hypotheses, decision thresholds, and outcome-conditional wording were fixed before any cell-type proportion was computed. The pre-registration document has SHA-256 digest 715cd5b6… and was locked at 2026-08-17T15:22:18Z; it is provided in full as Supplementary Material so that the registered predictions can be compared against the reported results. All derived results, analysis scripts, per-stage gate logs, and the SHA-256 manifest of archived artefacts are provided as Supplementary Material, permitting every numerical value reported in this paper to be traced to a specific output file without re-running read alignment.

## Supplementary Materials

The following supporting information can be downloaded at https://www.mdpi.com/article/10.3390/ani16203205/s1.
