# Supplementary material for: Gene-Length Bias and Compartment Non-Identifiability in Cell-Type Deconvolution of Sheep Skin: A Prospectively Specified and Timestamped Diagnostic Re-Analysis of Wool-Density Transcriptomes in Hetian Sheep
Source: Animals (Basel). 2026 Oct 12;16(20):3205. doi: 10.3390/ani16203205 (PMC13700806; doi:10.3390/ani16203205)
Supplement: Supplementary file 1 [file animals-16-03205-s001.zip › Reproducibility Information.pdf]

Reproducibility Information: Computational Environment, Data Sources, and Analysis Pipeline

Supplementary documentation prepared for the revision of the manuscript submitted to Animals (pre-registered computational re-analysis of PRJNA754683, Hetian sheep skin bulk RNA-seq, deconvolved against the ovine single-cell reference GSE186204). Compiled 30 September 2026. Every fact below is traceable to a specific archived file of the supplementary data inventory, named in the text.

1. Purpose and scope

This document responds to the request of the editor and reviewers for complete platform and experimental-process information supporting the computational re-analysis presented in the manuscript. It records (i) the GPU Server on which the whole study was executed, (ii) the full software environment with versions, (iii) the public data sources with access instructions and checksums, and (iv) the stage-by-stage analysis pipeline, including the version and primary methodological citation of every algorithm used. The aim is that an independent third party can re-run the entire analysis from the raw public data.

The analysis was performed once, end to end, on 17 August 2026 under a time-stamped pre-registration (Section 6). All bioinformatic computation, archived result integrity verification, manuscript assembly, and quality-control work were completed on the GPU Server. Every analysis stage wrote its own log, and no parameter was changed after results were observed, except where explicitly documented in the manuscript's Limitations section.

2. Compute platform and hardware

Hardware values below were verified on the server before the analysis began (allocation and parallel-scaling tests) and frozen again into the environment record at the end of Stage 0 on 17 August 2026, 16:48 UTC.

Item	Verified value
Role	All analysis stages 0–9 (both the bulk-RNA-seq track and the single-cell reference track); integrity verification of archived results; manuscript build; formatting and quality-control tasks.
CPU	AMD EPYC 7B13; 2 sockets x 64 cores; 256 logical threads

Item	Verified value
Parallel-scaling verification	Near-linear to 32 concurrent processes (16 processes: 15.5x speedup; 32 processes: 26.6x speedup)
RAM	976 GiB total; 24 GiB allocation test passed; no cgroup memory limit
Disk	2.8 TB free on NVMe storage; measured sequential write throughput 889 MB/s
GPU	NVIDIA GeForce RTX 4090; 24 GB; CUDA 13.0- provisioned on the machine but not required and not used by this project (see GPU statement below)
Operating system	Linux; kernel 6.8.0-83-generic (#83-Ubuntu SMP); x86_64
Hostname at run time	yotta-8hddahbu
Environment freeze	17 August 2026, 16:48 UTC (archived as host.txt)

GPU statement. Although the server carries an RTX 4090 GPU, the published analysis is entirely CPU-based: every analysis step (fastp, STAR, featureCounts, the R deconvolution stack, DESeq2, WGCNA, and the Scanpy/Leiden/Harmony single-cell processing) runs on CPU, and no GPU-accelerated computation appears anywhere in the pipeline. The Python environment records CUDA-enabled wheel packages (torch 2.11.0+cu128; CUDA toolkit 12.8.1) that were installed as part of the base stack but never invoked by any pipeline script. GPU hardware characteristics are therefore not part of the reproducibility-critical path; the GPU row in Table 1 comes from the pre-run hardware verification. This server is a high-performance machine with large memory and multi-core CPUs, far exceeding the capability of ordinary desktop computers.

3. Software environment

The complete environment was frozen at the end of Stage 0 (17 August 2026, 16:48 UTC) into four archival files that are part of the supplementary data inventory: host.txt (system and memory), tool_versions.txt (command-line tool versions), pip_freeze.txt (85 Python packages), and r_packages.txt (R packages, each load-tested). Package managers were micromamba (conda environments bioinf and renv) and pip. The global random seed was 20260817.

3.1 Base environment

Item	Value
Operating system	Linux; kernel 6.8.0-83-generic #83-Ubuntu SMP; x86_64 (Ubuntu distribution; the exact release string was not captured in the freeze, see Section 8)
Python	3.10.12 (micromamba environment bioinf)
R	4.4.3 (2025-02-28; micromamba environment renv)

Item	Value
Global random seed	20260817 (single seed for all stochastic steps)

3.2 Command-line tools used in the published pipeline

Tool	Version	Role in the published pipeline	Primary citation
fastp	0.23.4	Read QC and adapter trimming (Stage 2)	Chen S, Zhou Y, Chen Y, Gu J. 2018.
STAR	2.7.11b	Genome index and spliced alignment (Stage 3)	Dobin A, Davis CA, Schlesinger F, Drenkow J, Zaleski C, Jha S, Batut P, Chaisson M, Gingeras TR. 2013.
featureCounts (subread)	2.0.6	Gene-level read counting (Stage 3)	Liao Y, Smyth GK, Shi W. 2014.
samtools	1.21	BAM utilities in the alignment/counting stage (Stage 3)	Danecek P, Bonfield JK, Liddle J, et al. 2021
R	4.4.3	Statistical environment for Stages 4–9	R Core Team 2025. https://cran.r-project.org/
Python	3.10.12	Single-cell processing environment (Stages 4–5)	Python Software Foundation. https://www.python.org/
sra-tools	3.1.1	Installed as archive-access contingency; not needed (ENA delivery succeeded)	–
pigz	2.8	Parallel (de)compression	–

Tools installed and verified in the same environment but not invoked by any published analysis step: HISAT2 2.2.1, StringTie 2.2.3, PLINK 1.90b6.21, PLINK2, selscan 3.0.1, and liftOver; the Python packages pydeseq2 0.5.2 and shap 0.49.1; and the R packages edgeR 4.4.0, limma 3.62.1, Seurat 5.5.1, and randomForest/xgboost. This was verified by inspection of every pipeline script: none of these tools is called by a script that produced a published result. They were provisioned for contingency branches of the pre-approved plan that were not taken.

One environment fault occurred and was fixed inside Stage 0: the BayesPrism and MuSiC R packages initially failed to load after installation. The fault was diagnosed and repaired in Stage 0, before any deconvolution stage could depend on them, and the repaired installations were verified by loading each package and running its built-in test example. The failure, the fix, and the verification are documented in the Stage 0 log (s00.log).

3.3 R packages used in the published pipeline

Package	Version	Role
DESeq2	1.46.0	Differential expression, negative-binomial GLM (Stage 7)
WGCNA	1.74	Weighted gene co-expression network analysis (Stage 8)
MuSiC	1.0.0	Regression-based deconvolution (Stages 5-6)
SCDC	0.0.0.9000	Least-squares deconvolution with multiple single-cell references (Stages 5-6)
BayesPrism	2.2.3	Bayesian deconvolution (Stages 5-6)
nnls	1.6	Unregularised non-negative least squares (Stages 5-6)
clusterProfiler	4.14.0	GO and pathway enrichment (Stage 7)
SingleCellExperiment	1.28.0	Single-cell data container
Biobase	2.66.0	Expression-data container

3.4 Python packages used in the published pipeline

Package	Version	Role
scanpy	1.11.5	Single-cell processing: HVG selection, PCA, neighbourhood graph, marker scoring (Stage 4)
anndata	0.11.4	Single-cell data container (h5ad)
leidenalg	0.12.0	Leiden community detection (Stage 4)
igraph	1.0.0	Graph backend for Leiden
harmonypy	2.0.0	Batch correction across the two reference animals (Harmony reference variant)
umap-learn	0.5.12	UMAP embedding for cluster diagnostics
gseapy	1.3.1	Enrichment analysis (Stage 7)
numpy	2.2.6	Numerical base
scipy	1.15.3	Sparse matrices and statistics
pandas	2.3.3	Tabular data handling
h5py	3.16.0	HDF5 input/output
matplotlib	3.10.9	Figure rendering (Stage 9)
statsmodels	0.14.6	Statistical utilities
scikit-learn	1.7.2	General machine-learning utilities
torch 2.11.0+cu128; CUDA toolkit 12.8.1	–	Installed with the base stack; not invoked by any pipeline script (see GPU statement in Section 2)

3.5 Environment freeze artifacts

The freeze contains: host.txt (uname, thread count, memory; frozen 17 August 2026, 16:48 UTC); tool_versions.txt (one version line per command-line tool); pip_freeze.txt (85 Python packages with exact versions); r_packages.txt (R package versions, load-tested); and SEED.txt (the global seed 20260817). Conda environment exports for both environments (env_bioinf.yaml, env_renv.yaml) were also written at freeze time and are listed in the archive manifest.

4. Public data sources and download links

All input data are public. The three sources, their access points, and the integrity verification applied to each are listed in Table 3. The complete per-file FTP paths and MD5 values for all twelve FASTQ files, together with the post-download verification table, are part of the supplementary data inventory (ena_filereport.tsv; fastq_verified.tsv).

Dataset	Platform and accession	Download entry point	Contents and verification
Bulk skin RNA-seq; 6 Hetian sheep (3 dense-wool HS, 3 sparse-wool HP); source study Shi et al. 2022 (reference [28] of the manuscript)	ENA; PRJNA754683 (same project on NCBI: BioProject PRJNA754683, SRA)	https://www.ebi.ac.uk/ena/browser/view/PRJNA754683 (ENA browser; per-run FTP paths in the file report)	12 FASTQ files (6 runs x 2 mates; 150-bp paired-end; approx. 18.2 GB total). Downloaded from the ENA FTP mirror; every file MD5-verified against the ENA file report (12/12 PASS). Run-to-sample mapping: HS01=SRR15461556, HS02=SRR15461555, HS03=SRR15461554, HP01=SRR15461553, HP02=SRR15461552, HP03=SRR15461551.
Single-cell reference; ovine skin, day-3 lamb, one curly-wool and one straight-wool animal	GEO; GSE186204 (BioProject PRJNA772954)	https://www.ncbi.nlm.nih.gov/geo/query/acc.cgi?acc=GSE186204	Count matrix GSE186204_scRNA_seq_sheepskin.txt.gz (219,461,126 B), downloaded and size-verified; parsed to 14,037 cells x 18,161 genes; log1p(CP10K)

Dataset	Platform and accession	Download entry point	Contents and verification
			normalisation confirmed by expm1 row sums. Source publication: Wang S, Wu T, Sun J, Li Y, Yuan Z, Sun W. 2021. Single-cell transcriptomics reveals the molecular anatomy of sheep hair follicle heterogeneity and wool curvature. Frontiers in Cell and Developmental Biology 9:800157. doi:10.3389/fcell.2021.800157
Reference genome and annotation; Ovis aries	NCBI RefSeq; GCF_016772045.2 (ARS-UI_Ramb_v3.0)	https://ftp.ncbi.nlm.nih.gov/genomes/all/GCF/016/772/045/GCF_016772045.2_ARS-UI_Ramb_v3.0/ (dataset browser: https://www.ncbi.nlm.nih.gov/datasets/genome/GCF_016772045.2/)	genomic.fna.gz (MD5 faedef1d96083c2dd529609979ab14c0) and genomic.gtf.gz (MD5 005c33c878e3e6b92181c88bede302ad), verified against the NCBI md5checksums.txt; genome length approx. 2.654 Gb.

5. Analysis pipeline: steps, algorithms, and method citations

The pipeline comprised ten pre-defined stages (0-9) separated by hard quality gates; a stage could not start until the previous gate passed. Every stage wrote its own log, and one provenance row per stage (SHA-256 of the exact script, start and end times, exit code) was appended to PROVENANCE.tsv. The 21 pipeline scripts are provided in the supplementary data inventory; each documents its inputs, outputs, and gate criteria in comments. Total compute time was approx. 10-14 h at 32 threads. The

stages below give the algorithm, the key parameters, and the primary methodological citation for every step.

5.1 Stage 0 - Environment freeze and metadata

A single-source-of-truth sample table (six runs: three HS, three HP) was written once, with run-to-sample mapping and per-file MD5 checksums, and no analysis script was permitted to hard-code sample names. The environment was frozen (Section 3), the global seed was registered, and the deconvolution packages were repaired and load-verified.

5.2 Stage 1 - Raw-data acquisition and integrity verification

The twelve FASTQ files were downloaded from the ENA FTP mirror with resume-capable curl (at most four parallel streams; a segmented-fetch fallback handled slow files). Every file was MD5-verified against ENA's published checksums (12/12 PASS), and the FASTQ record structure and 150-bp read length were verified directly from the streams. Any checksum mismatch would have triggered re-download of that file only.

5.3 Stage 2 - Read quality control

fastp 0.23.4 (Chen et al. 2018, Bioinformatics) was run per library with paired-end adapter auto-detection, quality filtering (-q 15 -u 40, minimum length 50), and 8 threads, writing JSON and HTML reports. Aggregate QC gates: clean-read retention >= 90% and Q30 >= 85% per library; no library an outlier by more than two standard deviations. All six libraries passed.

5.4 Stage 3 - Reference, alignment, and quantification

The ARS-UI_Ramb_v3.0 assembly (GCF_016772045.2) was downloaded from NCBI and verified against md5checksums.txt (Table 3). A STAR 2.7.11b genome index was built with sjdbOverhang 149 (read length 150; Dobin et al. 2013, Bioinformatics), and each library was aligned with twopassMode Basic, outFilterMultimapNmax 20, outFilterMismatchNoverLmax 0.04, output as coordinate-sorted BAM. Gene-level counts were produced with featureCounts 2.0.6 (Liao et al. 2014, Bioinformatics): paired-end, --countReadPairs, exon-level, gene_id aggregation, unstranded (-s 0). Strandedness was determined empirically on one library by comparing assignment rates under -s 0/1/2 before the setting was applied to all six libraries. Alignment gates: uniquely mapped >= 80% per library; featureCounts assignment rate >= 60%.

5.5 Stage 4 - Reference-panel construction

The GSE186204 matrix (14,037 cells x 18,161 genes, log1p-CP10K) was processed with Scanpy 1.11.5 (Wolf et al. 2018, Genome Biology): 2,000 highly variable genes, 50 principal components, and Leiden clustering (Traag et al. 2019, Scientific Reports) at resolutions 0.4-1.5, in two parallel variants - uncorrected and Harmony-corrected (Korsunsky et al. 2019, Nature Methods) across the two reference animals. Clusters were assigned to seven pre-frozen compartments (hair-follicle epithelium; follicle-inducing mesenchyme DP+DS; dermal fibroblasts; interfollicular epidermis; hair-follicle stem cells; immune; endothelial) by marker-gene scoring with scanpy score_genes, recording the z-margin to the runner-up compartment. The assignment was cross-tabulated against the 16 labels published with the atlas (>= 80% consistency gate), and each compartment was required to be non-empty in both variants.

5.6 Stage 5 - Pseudo-bulk benchmark (algorithmic-correctness control)

To verify that the deconvolution implementation could recover known composition, the reference cells were split 50/50 with the fixed seed; one half served as the deconvolution reference and the other was aggregated into 30 pseudo-bulk mixtures with known, deliberately varied compartment proportions - including six mixtures that mimic the expected real contrast (approx. 8 percentage points in the hypothesis-bearing compartment, the magnitude implied by 33.79 vs 25.93 secondary follicles per mm2). All algorithms were run with exactly the code path, parameters, and preprocessing later used on the real data; recovery was scored by Pearson and Spearman correlation and RMSE against truth. Gate: at least two algorithms with overall Pearson r >= 0.80 and RMSE <= 0.10, and correct detection of the designed difference in at least five of the six mimic mixtures. Only algorithms passing this gate were used downstream.

5.7 Stage 6 - Deconvolution of the real libraries and pre-registered statistics

The six bulk count profiles were deconvolved with MuSiC (Wang et al. 2019, Nature Communications), SCDC (Dong et al. 2021, Briefings in Bioinformatics), BayesPrism (Chu et al. 2022, Nature Cancer), and unregularised non-negative least squares (R package nnls), in both reference variants. Hypothesis tests followed the pre-registration exactly: exact Wilcoxon rank-sum at n=3 vs 3, with Cliff's delta (Cliff 1993, Psychological Bulletin) as a non-parametric effect size; no regression or

multivariate modelling, which six samples cannot support. Pre-declared sensitivity analyses: 50 repeats of 50% reference sub-sampling; marker jackknife (dropping each compartment's markers in turn); and both batch variants. Gate: proportions sum to 1 for every sample and run; pairwise inter-algorithm agreement r >= 0.7 on compartment proportions.

5.8 Stage 7 - Differential expression

DESeq2 1.46.0 (Love et al. 2014, Genome Biology) was fitted on raw gene counts with design ~group (HS vs HP), and the design matrix rank was checked explicitly before any covariate model. Results were reported at the source study's threshold (|log2FC| > 0.585, q < 0.05) and a stricter threshold (|log2FC| > 1, padj < 0.05), and compared with the source study's DEG list, including the fate of its eight qPCR-validated genes. A composition-adjusted variant (~group + inferred compartment proportion) was run as an explicitly exploratory sensitivity check. GO and pathway enrichment used clusterProfiler and gseapy, before and after adjustment.

5.9 Stage 8 - WGCNA module-trait association

Variance-stabilised counts were filtered for low expression and analysed with WGCNA 1.74 (Langfelder and Horvath 2008, BMC Bioinformatics) as a signed network with blockwiseModules under the fixed seed. Soft-threshold power selection used the scale-free-topology criterion with an explicit slope safeguard, because at n = 6 the naive R-squared criterion is unreliable (powers whose fit had a positive slope were rejected). Module-trait correlations were computed against group (HS/HP) and against the inferred compartment proportions, every correlation being reported with its p-value and 4 degrees of freedom. Tree-based models and SHAP were deliberately not applied (n = 6 cannot support them).

5.10 Stage 9 - Figures and provenance audit

The five main figures were produced with each panel writing its own source CSV next to the image; supplementary tables were assembled. The provenance audit verified that PROVENANCE.tsv contains one row per stage with exit code 0 and that every archived file has an entry in MANIFEST.sha256. The archived snapshot was then transferred off the server and re-verified bit for bit (Section 6).

Algorithm or tool	Version	Stage	Primary citation
fastp (QC and trimming)	0.23.4	2	Chen S et al. 2018.
STAR (spliced aligner)	2.7.11b	3	Dobin A et al. 2013.

Algorithm or tool	Version	Stage	Primary citation
featureCounts (gene counting)	2.0.6	3	Liao Y et al. 2014.
Scanpy (single-cell processing)	1.11.5	4	Wolf FA et al. 2018.
Leiden community detection	leidenalg 0.12.0	4	Traag VA et al. 2019.
Harmony batch correction	harmonypy 2.0.0	4	Korsunsky I et al. 2019.
MuSiC deconvolution	1.0.0	5–6	Wang X, Park J, Susztak K, Zhang NR, Li M. 2019.
SCDC deconvolution	0.0.0.9000	5–6	Dong M et al. 2021.
BayesPrism deconvolution	2.2.3	5–6	Chu T, Wang Z, Pe'er D, Danko CG. 2022.
Non-negative least squares	nnls 1.6	5–6	R package nnls; algorithm of Lawson CL and Hanson RJ, Solving Least Squares Probems (1974)
Exact Wilcoxon rank-sum; Cliff's delta	base R stats	6	Cliff N. 1993.
DESeq2 (negative-binomial GLM)	1.46.0	7	Love MI, Huber W, Anders S. 2014.
WGCNA	1.74	8	Langfelder P, Horvath S. 2008.

6. Random seed, pre-registration, provenance, and integrity verification

Seed. A single global random seed (20260817) was fixed before the analysis, recorded in the environment freeze (SEED.txt), and consumed by every stochastic step: the seeded 50/50 cell split, the 50 sub-sampling repeats, Leiden initialisation, the BayesPrism sampler, and blockwiseModules.

Pre-registration. The analysis plan - the directional hypotheses (H1: hair-follicle epithelial proportion higher in HS than HP; H2: follicle-inducing mesenchyme proportion higher in HS than HP), the exact statistics, the three-branch reporting protocol, and all sensitivity analyses - was written and locked before any deconvolution ran: SHA-256 715cd5b636053ba96f44258423f7c0a2664bf3243eccf9643b59c1435aad1643, locked 17 August 2026, 15:22 UTC, about 36 minutes before Stage 0 began (15:58 UTC). The pre-registration file and its hash are part of the supplementary data inventory.

Provenance and integrity. PROVENANCE.tsv contains one row per stage with the SHA-256 of the exact script that ran, its start and end times, and its exit code.

MANIFEST.sha256 records the SHA-256 of every archived file. After the server was shut down, the complete archive - 227 files: pipeline scripts, stage logs, environment freeze, all intermediate result tables, figures, and metadata - was transferred off the server and re-verified bit for bit (227/227 checksums match). Every number reported in the manuscript traces to a file in this archive.

7. Resources required for independent reproduction

A third party reproducing the study end to end needs the following, all of which are public or included in the supplementary data inventory.

1. Hardware. Any machine with at least 64 GB RAM (the STAR index build peaks at approx. 40 GB) and about 100 GB of disk. The reference run used 32 threads on the GPU Server; total compute was approx. 10-14 h.
2. Software. The versions are listed in Tables 1-4 of Section 3 (Python 3.10.12, R 4.4.3, STAR 2.7.11b, fastp 0.23.4, featureCounts 2.0.6, and the R/Python package sets). The environment freeze files allow exact reconstruction; modest version drift in auxiliary packages is unlikely to affect results, but the frozen versions are the verified configuration.
3. Data. The three public sources in Section 4: approx. 18.2 GB of FASTQ (PRJNA754683), the 219 MB single-cell matrix (GSE186204), and the genome/annotation bundle (GCF_016772045.2). All checksums needed for verification are given in Section 4 and the supplementary inventory.
4. Code. The 21 pipeline scripts (Stages 0-9 plus installation and audit utilities), in which every input, output, and gate criterion is documented in comments.
5. Plan. The pre-registration document fixes the hypotheses, the statistics, the reporting branches, and the sensitivity analyses, so that a re-analysis can be compared stage by stage with the original.
6. Checkpoints. The gate criteria in Section 5 allow stage-by-stage verification: 12/12 FASTQ MD5 matches; >= 90% clean-read retention and >= 85% Q30; >= 80% uniquely mapped reads and >= 60% assignment rate; benchmark Pearson r >= 0.80 with RMSE <= 0.10 for at least two algorithms; proportions summing to 1 with inter-algorithm agreement r >= 0.7.

8. Documentation gaps

The following items could not be documented from the archived records and are reported as gaps rather than reconstructed: (i) the exact distribution release string of the server operating system - the freeze recorded the kernel identifier (6.8.0-83-generic, an Ubuntu kernel) but not /etc/os-release; (ii) post-analysis disk utilisation on the server - 2.8 TB free was verified at the pre-run hardware check, but

no disk measurement was frozen afterwards; (iii) no GPU model appears in the post-run environment freeze, because no GPU-accelerated step exists in the pipeline - the GPU specification in Table 1 comes from the pre-run hardware verification. All other facts in this document are traceable to named archived files.

The complete archive underlying this document - the pre-registration, the pipeline scripts, the stage logs, the environment freeze, and all intermediate result tables - is provided as the supplementary data inventory accompanying the manuscript.
