# Supplementary material for: Gene-Length Bias and Compartment Non-Identifiability in Cell-Type Deconvolution of Sheep Skin: A Prospectively Specified and Timestamped Diagnostic Re-Analysis of Wool-Density Transcriptomes in Hetian Sheep
Source: Animals (Basel). 2026 Oct 12;16(20):3205. doi: 10.3390/ani16203205 (PMC13700806; doi:10.3390/ani16203205)
Supplement: Supplementary file 1 [file animals-16-03205-s001.zip › supplementary_material.pdf]

Supplementary Material

Gene-Length Bias and Compartment Non-Identifiability in Cell-Type Deconvolution of Sheep Skin: a Pre-Registered Diagnostic Re-Analysis of Wool-Density Transcriptomes in Hetian Sheep

Supplementary Table S1.

Pre- versus post-gene-length-correction hair-follicle-epithelium proportion estimates across deconvolution algorithms and reference-processing variants.

**Deconvolution algorithm**	**Reference-processing variant**	**Compartment definition**	**Before gene-length correction (group mean, %)**	**After gene-length correction (group mean, %)**
**MuSiC**	No correction	Unmerged hair-follicle epithelium	3.3	81.4
**MuSiC**	Harmony corrected	Unmerged hair-follicle epithelium		
**SCDC**	No correction	Unmerged hair-follicle epithelium		
**SCDC**	Harmony corrected	Unmerged hair-follicle epithelium		
**BayesPrism**	No correction	Unmerged hair-follicle epithelium		
**BayesPrism**	Harmony corrected	Unmerged hair-follicle epithelium		
**NNLS**	No correction	Unmerged hair-follicle epithelium		
**NNLS**	Harmony corrected	Unmerged hair-follicle epithelium		
